# Face-Centered Cubic Refractory Alloys Prepared from Single-Source Precursors

**DOI:** 10.3390/ma13061418

**Published:** 2020-03-20

**Authors:** Kirill V. Yusenko, Saiana Khandarkhaeva, Maxim Bykov, Tymofey Fedotenko, Michael Hanfland, Alexander Sukhikh, Sergey A. Gromilov, Leonid S. Dubrovinsky

**Affiliations:** 1 BAM Federal Institute for Materials Research and Testing, Richard-Willstätter Str. 11, D-12489 Berlin, Germany; 2 Bayerisches Geoinstitut, Universität Bayreuth, D-95440 Bayreuth, Germany; Saiana.Khandarkhaeva@uni-bayreuth.de (S.K.); maks.byk@gmail.com (M.B.); timofeyfedotenko@gmail.com (T.F.); Leonid.Dubrovinsky@uni-bayreuth.de (L.S.D.); 3 ESRF – The European Synchrotron 71 Avenue des Martyrs, 38000 Grenoble, France; hanfland@esrf.fr; 4 Nikolaev Institute of Inorganic Chemistry, Lavrentiev ave. 3, 630090 Novosibirsk, Russia; a_sukhikh@niic.nsc.ru (A.S.); grom@niic.nsc.ru (S.A.G.)

**Keywords:** refractory alloys, platinum group metals, single-source precursors, high-pressure

## Abstract

Three binary *fcc*-structured alloys (*fcc*–Ir_0.50_Pt_0.50_, *fcc*–Rh_0.66_Pt_0.33_ and *fcc*–Rh_0.50_Pd_0.50_) were prepared from [Ir(NH_3_)_5_Cl][PtCl_6_], [Ir(NH_3_)_5_Cl][PtBr_6_], [Rh(NH_3_)_5_Cl]_2_[PtCl_6_]Cl_2_ and [Rh(NH_3_)_5_Cl][PdCl_4_]·H_2_O, respectively, as single-source precursors. All alloys were prepared by thermal decomposition in gaseous hydrogen flow below 800 °C. *Fcc*–Ir_0.50_Pt_0.50_ and *fcc*–Rh_0.50_Pd_0.50_ correspond to miscibility gaps on binary metallic phase diagrams and can be considered as metastable alloys. Detailed comparison of [Ir(NH_3_)_5_Cl][PtCl_6_] and [Ir(NH_3_)_5_Cl][PtBr_6_] crystal structures suggests that two isoformular salts are not isostructural. In [Ir(NH_3_)_5_Cl][PtBr_6_], specific Br…Br interactions are responsible for a crystal structure arrangement. Room temperature compressibility of *fcc*–Ir_0.50_Pt_0.50_, *fcc*–Rh_0.66_Pt_0.33_ and *fcc*–Rh_0.50_Pd_0.50_ has been investigated up to 50 GPa in diamond anvil cells. All investigated *fcc*-structured binary alloys are stable under compression. Atomic volumes and bulk moduli show good agreement with ideal solutions model. For *fcc*–Ir_0.50_Pt_0.50_, *V*_0_/*Z* = 14.597(6) Å^3^·atom^−1^, *B*_0_ = 321(6) GPa and *B*_0_’ = 6(1); for *fcc*–Rh_0.66_Pt_0.33_, *V*_0_/*Z* = 14.211(3) Å^3^·atom^−1^, *B*_0_ =259(1) GPa and *B*_0_’ = 6.66(9) and for *fcc*–Rh_0.50_Pd_0.50_, *V*_0_/*Z* = 14.18(2) Å^3^·atom^−1^, *B*_0_ =223(4) GPa and *B*_0_’ = 5.0(3).

## 1. Introduction

Traditionally, high-entropy alloys were prepared using conventional melting of pure metals. Nevertheless, catalytic applications as well as preparation of high-entropy alloys based on metals with ultra-high melting points need the development of new techniques for a preparation of high-entropy alloys as fine nanostructured powders. Recently, single-source precursors strategy has been applied to access high-entropy alloys based on platinum group metals. The strategy requires a synthesis of coordination compounds from water solutions and their further thermal decomposition in a hydrogen flow based on the following principle general scheme [1]:

in water solution: *a*[Ir(NH_3_)_5_Cl]Cl_2_ + *b*[Rh(NH_3_)_5_Cl]Cl_2_ + (1-*a-b*)[Ru(NH_3_)_5_Cl]Cl_2_ + *c*(NH_4_)_2_[IrCl_6_] + *d*(NH_4_)_2_[OsCl_6_] + *e*(NH_4_)_2_[PtCl_6_] + (1-*c-d-e*)(NH_4_)_2_[ReCl_6_] →

[Ir(NH_3_)_5_Cl]*_a_*[Rh(NH_3_)_5_Cl]*_b_*[Ru(NH_3_)_5_Cl]_(1-*a-b*)_[IrCl_6_]*_c_*[OsCl_6_]*_d_*[PtCl_6_]*_e_*[ReCl_6_]_(1-*c-d-e*)_ + 2NH_4_Cl;

in solid state: [Ir(NH_3_)_5_Cl]*_a_*[Rh(NH_3_)_5_Cl]*_b_*[Ru(NH_3_)_5_Cl]_(1-*a*-*b*)_[IrCl_6_]*_c_*[OsCl_6_]*_d_*[PtCl_6_]*_e_*[ReCl_6_]_(1-*c*-*d*-*e*)_ +7/2H_2*(gas)*_ → Ir_0.5(*a*+*c*)_Rh_0.5*b*_Ru_0.5(1-*a*-*b*)_Os_0.5*d*_Pt_0.5*e*_Re_0.5(1-*c*-*d*-*e*)_ + 5NH_4_Cl*_(gas)_* + 2HCl*_(gas)_*.

Several two phases as well as *hcp*–Ir_0.19_Os_0.22_Re_0.21_Rh_0.20_Ru_0.19_ and *fcc*–Ir_0.26_Os_0.05_Pt_0.31_Rh_0.23_Ru_0.15_ single-phase high-entropy alloys were prepared using the single-source precursors strategy [1]. The approach is quite general and can be extended to access refractory high-entropy alloys in a broad compositional range. Single-phase *fcc*- and *hcp*-structured high-entropy alloys were also tested under extreme conditions to characterize their pressure and temperature stability. Nevertheless, due to their compositional complexity, a little was investigated regarding a mechanism of their formation from single source precursors.

Refractory multicomponent alloys such as platinum group alloys attract attention as catalytically active species with high mechanical and chemical stability. Ir- and Rh-based alloys were proposed as materials for high-temperature applications, as thermocouples and crucibles. Refractory alloys usually have also high stability under extreme conditions and show low compressibility. Their low compressibility might be compared only with diamond [2,3,4]. Previously, several binary and multicomponent refractory systems of *fcc*- and *hcp*-structured alloys were investigated under extreme conditions [5,6,7,8]. It has been shown that all known platinum group alloys do not show any temperature and/or pressure induced phase transitions. Such a finding makes platinum group alloys important as stable materials with a unique phase and structural stability under extreme conditions. Ultra-incompressible alloys with Re, Os (*hcp*) and Ir (*fcc*) were investigated in detail up to 140 GPa; nevertheless, compressibility of *fcc*-structured alloys of metals with high compressibility such as Rh and Pd were not investigated so far. Among *fcc*-structured alloys, only *fcc*–Ir_0.42_Rh_0.58_ has been investigated up to 57 GPa at room temperature [5].

In the current study, we report synthesis of *fcc*–Ir_0.50_Pt_0.50_, *fcc*–Rh_0.66_Pt_0.33_ and *fcc*–Rh_0.50_Pd_0.50_ binary alloys from [Ir(NH_3_)_5_Cl][PtCl_6_], [Ir(NH_3_)_5_Cl][PtBr_6_], [Rh(NH_3_)_5_Cl]_2_[PtCl_6_]Cl_2_ and [Rh(NH_3_)_5_Cl][PdCl_4_]·H_2_O, respectively, as single-source precursors under low temperature. Investigation of *fcc*–Ir_0.50_Pt_0.50_, *fcc*–Rh_0.66_Pt_0.33_ and *fcc*–Rh_0.50_Pd_0.50_ binary alloys under hydrostatic compression up to 50 GPa in diamond anvil cells allow us to obtain bulk moduli for *fcc*-structured binary alloys. Equations of state, thermal expansion and pressure compressibility for known refractory high-entropy alloys can be validated with experimental data obtained for *fcc*-structured refractory binaries.

## 2. Materials and Methods

[Ir(NH_3_)_5_Cl]Cl_2_ and [Rh(NH_3_)_5_Cl]Cl_2_ were prepared from IrCl_4_·*x*H_2_O and RhCl_3_·*x*H_2_O according to published protocols [9,10]. (NH_4_)_2_[PtCl_6_], (NH_4_)_2_[PdCl_4_] and (NH_4_)_2_[IrCl_6_] were obtained from (Sigma Aldrich). Binary alloys *fcc*–Ir_0.50_Pt_0.50_ (sample A), *fcc*–Rh_0.66_Pt_0.33_ (sample B) and *fcc*–Rh_0.50_Pd_0.50_ (sample C) were prepared from [Ir(NH_3_)_5_Cl][PtCl_6_], [Rh(NH_3_)_5_Cl]_2_[PtCl_6_]Cl_2_ and [Rh(NH_3_)_5_Cl][PdCl_4_]·H_2_O respectively. Details about the synthesis of solid-state precursors and alloys can be found in our earlier publications [8,10]. Briefly, the precursors, [Ir(NH_3_)_5_Cl][PtCl_6_], was first crystallized at room temperature from a mixture of water solutions of [Ir(NH_3_)_5_Cl]Cl_2_ and (NH_4_)_2_[PtCl_6_], filtered and dried on air. The obtained solid powder of [Ir(NH_3_)_5_Cl][PtCl_6_] was then heated in the hydrogen flow up to 700 °C and cooled down to ambient temperature. Similarly, [Rh(NH_3_)_5_Cl][PdCl_4_]·H_2_O was prepared at room temperature from [Rh(NH_3_)_5_Cl]Cl_2_ and (NH_4_)_2_[PdCl_4_] and heated in hydrogen flow to 400 °C and cooled to ambient temperature during 2 h. [Rh(NH_3_)_5_Cl]_2_[PtCl_6_]Cl_2_ was prepared from water solution of (NH_4_)_2_[PtCl_6_] in 0.1 M HCl and solid [Rh(NH_3_)_5_Cl]Cl_2_. The mixture was kept in darkness at room temperature for a week. Orange crystals were filtered and dried on air. [Rh(NH_3_)_5_Cl]_2_[PtCl_6_]Cl_2_ crystals were decomposed in hydrogen flow at 700 °C. Similarly, all compounds were also decomposed in He flow. All preparatory conditions for all alloys are summarized in Table 1.

Two isoformular salts, [Ir(NH_3_)_5_Cl][PtCl_6_] and [Ir(NH_3_)_5_Cl][PtBr_6_], were prepared by the mixing of hot water solutions of [Ir(NH_3_)_5_Cl]Cl_2_ and (NH_4_)_2_[PtCl_6_] or (NH_4_)_2_[PtBr_6_], respectively. After 40–60 min, orange precipitate of [Ir(NH_3_)_5_Cl][PtCl_6_] and orange-red precipitate of [Ir(NH_3_)_5_Cl][PtBr_6_] were filtered, washed with minimum water, ethanol and dried on air. Red plate-shaped single crystals of [Ir(NH_3_)_5_Cl][PtBr_6_] were collected from the whole portion of the sample. Yield for [Ir(NH_3_)_5_Cl][PtCl_6_] was 75%–80% and yield for [Ir(NH_3_)_5_Cl][PtBr_6_] was 85%–90%.

Elemental analysis:
**Composition****Ir+Pt, wt %, Calculated****Ir+Pt, wt %, Obtained**[Ir(NH_3_)_5_Cl][PtBr_6_]39.2340.3 ± 0.2[Ir(NH_3_)_5_Cl][PtCl_6_]53.7553.3 ± 0.2

**Figure 1:**
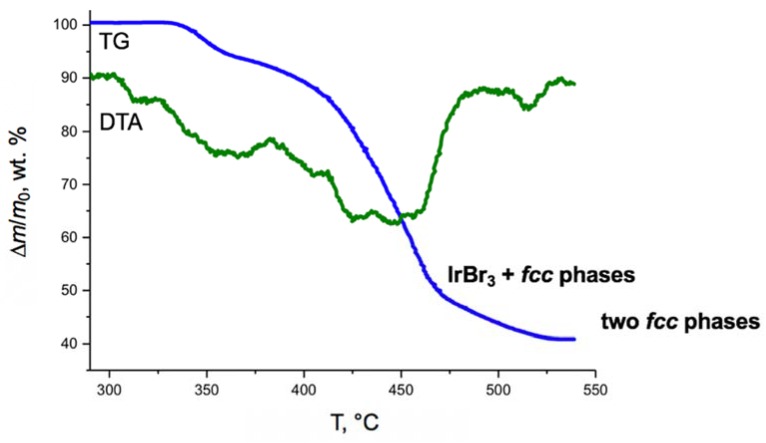
Thermal gravimetry (TG) and differential thermal analysis (DTA) curves for [Ir(NH_3_)_5_Cl][PtBr_6_] in helium flow (closed Pt crucible, 5 K/min, 150 mL/min).

Thermal analysis of [Ir(NH_3_)_5_Cl][PtCl_6_] and [Ir(NH_3_)_5_Cl][PtBr_6_] was performed on a Q–1000 TG device. Powders (*ca*. 0.1 g) were heated in Pt crucibles closed with a lead. Heating (5 K/min) was performed in helium flow (150 mL/min). Al_2_O_3_ has been used as a reference. [Ir(NH_3_)_5_Cl][PtCl_6_] decomposes in a narrow temperature interval 325–425 °C; [Ir(NH_3_)_5_Cl][PtBr_6_] decomposes between 330 and 530 °C (Figure 1).

Phase composition and cell parameters of metallic alloys were obtained by in house powder X-ray diffraction (PXRD) using an ARL X’TRA diffractometer (Cu*K*α-radiation, Ni-filter, position sensitive detectors, Bragg–Brentano reflection geometry, 2Θ = 5–100°, Δ2Θ = 0.03°, 10 s/step, room temperature, Thermo Electron Corporation, Waltham, MA, USA). Polycrystalline samples were slightly ground with hexane using an agate mortar, and the resulting suspensions were deposited on the polished side of a quartz sample holder, a smooth thin layer formed after drying. Silicon powder was taken as an external standard (*a* = 5.4309 Å, full width at half maximum 2Θ = 0.1°) for the calibration of the zero-shift of the goniometer and instrumental line broadening. Only single- and two-phase *fcc*-structured alloys were found as products of thermal decomposition of single-source precursors mentioned above (Table 1).

Scanning electron microscopy (SEM) images for *fcc*–Ir_0.50_Pt_0.50_, *fcc*–Rh_0.66_Pt_0.33_ and *fcc*–Rh_0.50_Pd_0.50_ binary alloys prepared in hydrogen atmosphere were obtained on XL30 ESEM (Environmental Scanning Electron Microscope) from FEI (Thermo Fisher Scientific, Waltham, Massachusetts, USA). The composition was obtained by energy-dispersive X-ray analysis (EDAX, equipped with Si-(Li) detector) and averaged for 5–6 points. The averaged composition was close to the nominal composition of single-source precursors. So, *fcc*–Ir_0.509(5)_Pt_0.491(5)_ correspond to *fcc*–Ir_0.50_Pt_0.50_ (sample A); *fcc*–Rh_0.657(4)_Pt_0.343(4)_ correspond to *fcc*–Rh_0.66_Pt_0.33_ (sample B); and *fcc*–Rh_0.497(5)_Pd_0.502(5)_ correspond to *fcc*–Rh_0.50_Pd_0.50_ (sample C).

Room temperature compressibility curves for *fcc*–Ir_0.50_Pt_0.50_, *fcc*–Rh_0.66_Pt_0.33_ and *fcc*–Rh_0.50_Pd_0.50_ binary alloys were collected at the ID15B beamline up to 40 GPa (ESRF, λ = 0.411235 Å, MAR 555 flat panel detector, beam size 10(*v*) × 10(*h*) μm^2^). The samples were loaded in diamond anvil cells equipped with conically supported Boehler Almax anvils 250 μm culet sizes. He was used as a pressure transmitting medium. Ruby was applied as a pressure calibrant. The diffraction data were integrated using DIOPTAS [17]. The unit cell parameters, the background and the line-profile parameters were refined simultaneously using JANA2006 software [18]. The *P*–*V* data were fitted using EoS–Fit 5.2 software [19].

The X-ray diffraction study of [Ir(NH_3_)_5_Cl][PtBr_6_] single crystal at 150 K was performed on an automated Bruker APEX-II CCD diffractometer (MoKα radiation, graphite monochromator, two-dimensional CCD detector, Bruker Corporation, Karlsruhe, Germany). One hundred and fifty nine structural parameters with 48 restrains were refined and 4159 reflexes were used. The corresponding divergence factors were *R*_all_ = 9.07% and *wR*_ref_ = 15.41%; for 3150 reflections with *I* ≥ 2σ(*I*), *R*_gt_ = 6.28%, *wR*_gt_ = 14.33% and the *S* factor against *F*_2_ was 1.066. X-ray crystallographic data were deposited with an Inorganic Crystal Structure Database (ICSD) under No. 1971298.

## 3. Results and Discussion

### 3.1. Crystal Structures of [Ir(NH_3_)_5_Cl][PtCl_6_] and [Ir(NH_3_)_5_Cl][PtBr_6_] Single-Source Precursors

Synthesis and crystal structures for isoformular [*M*^I^(NH_3_)_5_Cl]_2_[*M*^II^Cl_6_]Cl_2_ compounds where *M*^I^ = Co, Cr, Ir, Rh, Os and *M*^II^ = Ir, Pt, Re, Os as well as for [*M*^I^(NH_3_)_5_Cl][*M*^II^*Г*_4_] with *M*^I^ = Co, Cr, Ir, Rh and Os, *M*^II^ = Pt and Pd, *Г* = Cl and Br were described for all possible metallic combinations [20,21]. Nevertheless, not all [*M*^I^(NH_3_)_5_Cl][*M*^II^*Г*_6_] compounds *M*^I^ = Co, Cr, Ir, Rh and Os, *M*^II^ = Ir, Pt, Re and Os and Г = Cl and Br were synthetized and structurally investigated so far. For [*M*^I^(NH_3_)_5_Cl]_2_[*M*^II^Cl_6_]Cl_2_ and [*M*^I^(NH_3_)_5_Cl][*M*^II^*Г*_4_], it has been shown that isoformular compounds were isostructural and could be co-crystallized from water solutions. [*M*^I^(NH_3_)_5_Cl][*M*^II^Cl_6_] salts were crystallized in the *P*2_1_/*m* space group. All species were also isostructural with very close cell parameters.

[*M*^I^(NH_3_)_5_Cl][*M*^II^Br_6_] compounds were synthetized only for selected metals. It has been shown that various metals in anion give various types of crystal structures. To clarify general trends in the isoformular compounds where not all members are isostructural is was important to extend them with other examples. In the current study, [Ir(NH_3_)_5_Cl][PtBr_6_] salt was prepared as single crystals and its crystal structure was compared with isoformular [Ir(NH_3_)_5_Cl][PtCl_6_], [Rh(NH_3_)_5_Cl][PtBr_6_], [Ir(NH_3_)_5_Cl][PtBr_6_] and [Rh(NH_3_)_5_Cl][IrBr_6_] and also previously structurally characterized by X-ray diffraction as single crystals (Table 2).

All investigated isoformular double complex salts contain isolated chloropentammine cations (Rh and Ir) with local symmetry *m* with one NH_3_ and one Cl ligand sitting in the mirror plane. Hexachloro- and hexabromometalates(IV) as anions had local symmetry 2/*m* with four Cl-ligands placed in the mirror-plane. In all crystal structures, six anions have six neighboring cations as well as six cations had six neighboring anions. The coordination polyhedral for cations and anions was deformed octahedra, so general crystal structures can be described as deformed NaCl structures with closed packing of isometric cations and anions. Cations and anions form regular closed-packed hexagonal layers perpendicular to the direction [110].

**Figure 2:**
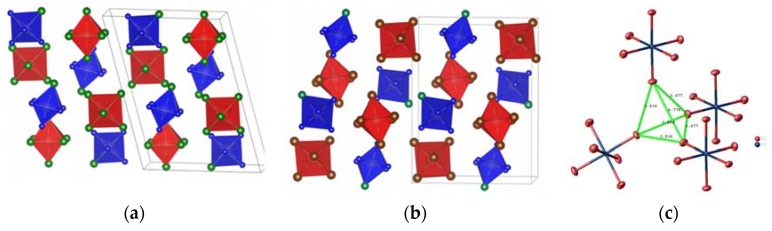
Crystal structure of [Rh(NH_3_)_5_Cl][OsCl_6_] along the monoclinic axis *y* (**a**) [14]; right: crystal structure of [Ir(NH_3_)_5_Cl][PtBr_6_] along the monoclinic axis *y* and (**b**) ([Rh(NH_3_)_5_Cl]^2+^ or [Ir(NH_3_)_5_Cl]^2+^ cations are shown in red, [OsCl_6_]^2−^ or [PtBr_6_]^2-^ anions and N – in blue, green – Cl or Br; H atoms are omitted for clarity); short Br…Br contacts between [PtBr_6_]^2-^ anions in the [Ir(NH_3_)_5_Cl][PtBr_6_] crystal structure (**c**).

Salts with hexachlorometallates(IV) as anions had relatively low solubility and were difficult in crystallization from water solution. Only parent compound [Rh(NH_3_)_5_Cl][OsCl_6_] was crystallized as a single crystal (Figure 2) [14]. [Ir(NH_3_)_5_Cl][PtCl_6_] was obtained only as crystalline powder. Its crystal structures were similar to isoformular [Rh(NH_3_)_5_Cl][PtCl_6_] with close cell parameters.

Double complex salts with hexabromometallates(IV) as anions have usually a higher solubility and can be prepared as single crystals suitable for X-ray diffraction study [15]. Crystal structures of [Rh(NH_3_)_5_Cl][IrBr_6_] and [Rh(NH_3_)_5_Cl][PtBr_6_] were described in [12,15] based on single crystal X-ray diffraction data. Salts with hexabromometallates(IV) as anions have monoclinic structures solved in similar to the haxachlorometallates(IV) *P*2_1_/*m* space group. Nevertheless, monoclinic angle β was close to 90°. [Ir(NH_3_)_5_Cl][PtBr_6_] had the same crystal structure as [Rh(NH_3_)_5_Cl][PtBr_6_] with a monoclinic angle much closer to 90°. Due to the monoclinic angle, all selected single crystals were twinned by 180° rotation around the *c* axis and might be indexed as orthorhombic. Diffraction reflections were much blurrier at higher angles. Nevertheless, its crystal structure was not orthorhombic and did not show higher symmetry. Tests for higher possible symmetry did not give positive results. The corresponding dataset for [Ir(NH_3_)_5_Cl][PtBr_6_] was collected and integrated in the orthorhombic crystal system and later refined as a two component twin with 0.528/0.472 twin fractions. The real value of the monoclinic angle was approximately 90.5° at 150 K and 90.8° at 90 K (estimated using 100 weak individual reflections at high diffraction angles).

Crystal structures of [Ir(NH_3_)_5_Cl][PtBr_6_] and [Rh(NH_3_)_5_Cl][PtBr_6_] were similar to chloride analogous. Hexagonal closed packed layers of cations and anions typical for chloride structures were broken due to the presence of several short Br…Br (3.681, 3.831, 3.838 Å) and Br…Cl (3.533 Å) contacts. In [Ir(NH_3_)_5_Cl][PtBr_6_], similar short contacts could be also found. Such Br…Br contacts were responsible for the packing of anions in the crystal structure as it was shown for other structures with hexabromometallates (IV).

### 3.2. Preparation of fcc-Structured Binary Alloys under Ambient Pressure and Their Phase Composition

All *fcc*-structured alloys were prepared from single-source precursors (Table 1). Among *fcc*-structured binary metallic systems, only the Pt–Rh pair has complete miscibility in the solid state [22]. In all other binaries, there are miscibility gaps between two *fcc*-structured alloys: for Ir–Rh below 1335 °C; for Pd–Rh below 910 °C and for Ir–Pt below 1370 °C [23,24,25]. Nevertheless, all single-source precursors gave single-phase alloys as products of their thermal decomposition in a reductive atmosphere even below 700 °C (Table 1). Thermal decomposition in an inert atmosphere usually results in a formation of two-phase mixtures, which might be a sign for different decomposition mechanisms.

All binary systems allow us to prepare various alloys by changing compositions of single-source precursors. So, crystallization of [*M*^I^(NH_3_)_5_Cl]^2+^ (*M*^I^ = Rh, Ir) with [*M*^II^Cl_6_]^2-^ (*M*^II^ = Ir, Pt) from neutral water solutions results in a formation of [*M*^I^(NH_3_)_5_Cl][*M*^II^Cl_6_], which can be used as precursors for *fcc*–*M*^I^_0.5_*M*^II^_0.5_ alloys: [Rh(NH_3_)_5_Cl][IrCl_6_] for *fcc*–Ir_0.5_Rh_0.5_; [Rh(NH_3_)_5_Cl][PtCl_6_] for *fcc*–Rh_0.5_Pt_0.5_; [Ir(NH_3_)_5_Cl][PtCl_6_] for *fcc*–Ir_0.5_Pt_0.5_. Similar crystallization from HCl-containing solutions results in a formation of [*M*^I^(NH_3_)_5_Cl]_2_[*M*^II^Cl_6_]Cl_2_, which can be used as precursors for *fcc*–*M*^I^_0.66_*M*^II^_0.33_ alloys: [Rh(NH_3_)_5_Cl]_2_[IrCl_6_]Cl_2_ for *fcc*–Ir_0.66_Rh_0.33_; [Rh(NH_3_)_5_Cl]_2_[PtCl_6_]Cl_2_ for *fcc*–Rh_0.66_Pt_0.33_ and [Ir(NH_3_)_5_Cl]_2_[PtCl_6_]Cl_2_ for *fcc*–Ir_0.66_Pt_0.33_. Only Ir–Pd and Rh–Pd systems allowed only a single type of precursors: [Ir(NH_3_)_5_Cl][PdCl_4_] and [Rh(NH_3_)_5_Cl][PdCl_4_] for *fcc*–Ir_0.5_Pd_0.5_ and *fcc*–Rh_0.5_Pd_0.5_ alloys respectively.

It seems that thermal decomposition of described systems could be controlled by a reaction atmosphere. In the reductive flow (hydrogen), all systems Ir–Rh, Pd–Rh, Ir–Pt and Pt–Rh formed single phase *fcc*-structured alloys. Single-phase alloys formed in systems with and without miscibility in the solid-state. In the inert atmosphere (argon or helium flow), Ir–Rh forms also a single-phase *fcc*-alloy [5]. Ir–Pt and Pt–Rh (both systems with miscibility in the solid-state) formed a two-phase mixture after heating in an inert flow, which might be due to the mechanism of their thermal decomposition.

In an inert atmosphere (He, Ar and N_2_), hexachlorometallates(IV) decompose in a relatively narrow temperature interval [20,21]. For [Rh(NH_3_)_5_Cl][PtBr_6_] and [Rh(NH_3_)_5_Cl][PtBr_4_] [12,26], it has been shown that their thermal decomposition in an inert atmosphere corresponds to the formation of metallic *fcc*–Pt and RhBr_3_ as intermediates above 500 °C. Further heating results in the decomposition of RhBr_3_ and formation of two-phase *fcc*–alloys mixture. Such a transformation is a key process responsible for the formation of two-phase metallic products in an inert gas flow. Similarly, upon heating of [Ir(NH_3_)_5_Cl][PtBr_6_] above 500 °C intermediate with a total composition of “Ir:Pt:Br” contains broad reflexes corresponding to IrBr_3_ and two *fcc*–structured alloys:

[Ir(NH_3_)_5_Cl][PtBr_6_] → IrBr_3_ + “mixture of *fcc*-structured alloys“.

Further heating results in the formation of a two-phase metallic mixture (Table 1). In general, thermal decomposition of [*M*^I^(NH_3_)_5_Cl][*M*^II^Br_6_] occurred at higher temperatures in comparison with chloride [*M*^I^(NH_3_)_5_Cl][*M*^II^Cl_6_] analogous. Their thermal decomposition was overcome through the formation of rhodium or iridium bromides as intermediates, which was responsible for the formation of two-phase mixtures as final products of their thermal decomposition in an inert atmosphere. Thermal decomposition of [Ir(NH_3_)_5_Cl][PtCl_6_] and [Ir(NH_3_)_5_Cl][PtBr_6_] started at the same temperature (325 and 330 °C, correspondently), which could be due to the nature of their cation. Probably their thermal decomposition starts from the destruction of [Ir(NH_3_)_5_Cl]^2+^ cation.

**Figure 3:**
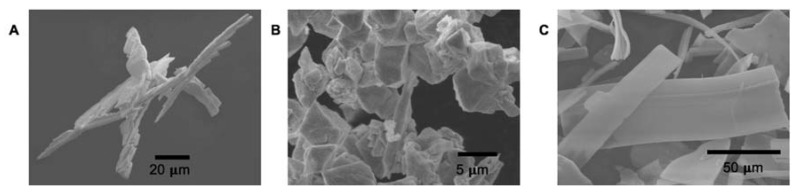
SEM images for *fcc*–Ir_0.50_Pt_0.50_ (**A**), *fcc*–Rh_0.66_Pt_0.33_ (**B**) and *fcc*–Rh_0.50_Pd_0.50_ (**C**) binary alloys.

Single-phase alloys prepared from single-source precursors show isometric porous metallic particles (Figure 3). The shape of porous conglomerates followed the shape of crystals characteristic for single source precursors.

### 3.3. High-Pressure Compressibility of fcc-Structured Binary Refractory Alloys

**Figure 4:**
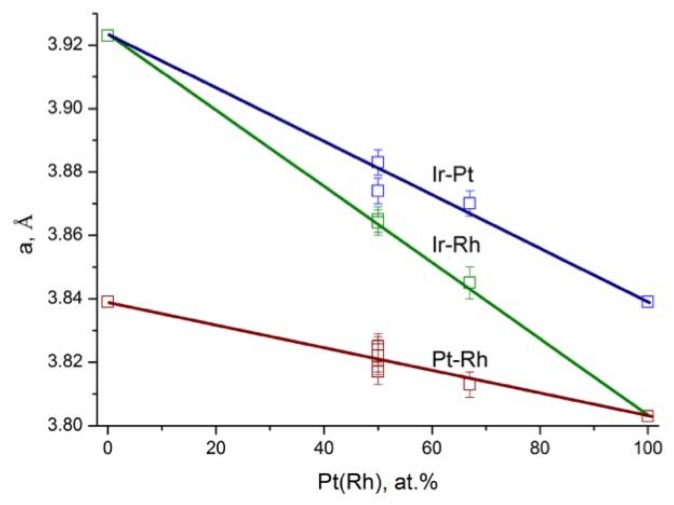
Dependence of cell parameters on composition for *fcc*-structured Rh, Ir, Pt and Pd single-phase binary alloys (according to Table 1).

Cell parameters characteristic for prepared *fcc*-alloys correspond to Zen’s low [27,28] and nearly linearly depend on the alloy’s compositions (Figure 4). Within experimental errors there is no positive or negative deviation from linearity, which might be a sign for ideality of described *fcc*-structured binary alloys. For *fcc*-structured binary alloys (and also for *hcp*-structured) with *hcp* metals such as Ir–Re and Rh–Re alloys, significant negative deviation from linearity has been mentioned [29,30].

**Figure 5:**
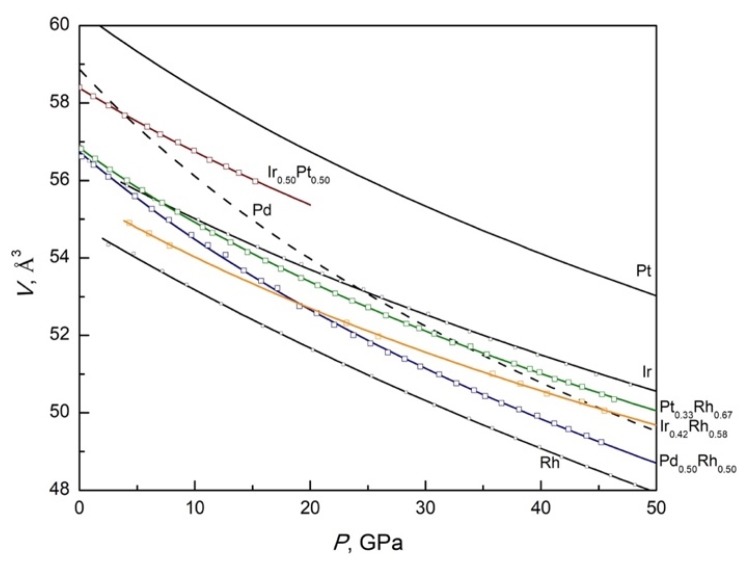
Room temperature high-pressure compressibility curves for *fcc*-structured Rh, Ir, Pt and Pd binary alloys and pure metals in *V*/*Z* vs. *P* scale (according to Table 3).

All investigated *fcc*-structured refractory alloys do not show any pressure-induced phase transitions below 50 GPa at room temperature. A similar pressure temperature stability was obtained for pure Rh, Ir, Pd and Pt up to much higher pressures. Experimental compressibility curves (*P*–*V* data) for all investigated alloys can be fitted using the third-order Birch-Murnaghan equation of state (BM–EoS) [31,32] (Table 3, Figure 5):

(1) $$P\left(V\right)=\frac{3B_{0}}{2}\left[{\left(\frac{V_{0}}{V}\right)}^{\frac{7}{3}}-{\left(\frac{V_{0}}{V}\right)}^{\frac{5}{3}}\right]\left\{1+\frac{3}{4}\left(B_{0}^{’}-4\right)\left[{\left(\frac{V_{0}}{V}\right)}^{\frac{2}{3}}-1\right]\right\},$$

where *V*_0_ is the unit cell volume at ambient pressure, *B*_0_ is the bulk modulus and *B’*_0_ is the pressure derivative of the bulk modulus. All alloys show regular compressibility with pressure as well as with composition.

It has been previously shown that bulk moduli for binary alloys can be estimated using quite a simple model reported in [5,6,8]. Concentration dependence of the bulk modulus *B*_0_(*x*) of a binary metallic alloy *M*^1^*_x_M*^2^_1-*x*_ containing *x* atomic fraction of the refractory metal can be calculated using the following equation:

(2) $$B_{0}\left(x\right)=B_{2}\left[\frac{1+x\left(\frac{V_{1}}{V_{2}}-1\right)}{1+x\left(\frac{B_{2}V_{1}}{B_{1}V_{2}}-1\right)}\right]$$

where *B*_1_ and *B*_2_ (GPa) are the bulk moduli of the metals *M*^1^ and *M*^2^, and *V*_1_ and *V*_2_ (Å^3^) are atomic volumes at ambient pressure of *M*^1^ and *M*^2^, correspondently. According to Table 3, structural parameters (atomic volume and bulk moduli) for all alloys can be estimated quite well using simple models typical for ideal solid solutions. Such a finding can be used for a prediction of the compressibility of new alloys to be able to construct a complete thermodynamic database for refractory *fcc*-alloys.

Palladium shows relatively high compressibility in the comparison with the other platinum group metals. As a result, the *fcc*–Rh_0.50_Pd_0.50_ alloy had the highest compressibility among other alloys. At the same time its compressibility had the better accordance with the ideal solution model (Equation (2)). A similar good satisfaction between experimentally obtained data and predicted according to Equation (2) were found for the *fcc*–Ir_0.42_Rh_0.58_ alloy. Both investigated platinum alloys *fcc*–Ir_0.50_Pt_0.50_ and *fcc*–Pt_0.33_Rh_0.67_ show large deviations from predicted values. A larger value for *fcc*–Ir_0.50_Pt_0.50_ can be explained by relatively low experimental pressure. Nevertheless, compressibility of *fcc*–Pt_0.33_Rh_0.67_ was investigated up to 47 GPa. Its compressibility was much higher in comparison with ideal solutions model. The mentioned large deviation from the ideal solutions model should be further studied theoretically.

Single-phase refractory high-entropy alloys, namely *hcp*–Ir_0.19_Os_0.22_Re_0.21_Rh_0.20_Ru_0.19_ and *fcc*–Ir_0.26_Os_0.05_Pt_0.31_Rh_0.23_Ru_0.15_, prepared from single-source precursors show similar numbers for atomic volumes and room-temperature compressibility (Table 3). Their behavior suggests that they can be described as ideal solid solutions [1,5]. Prepared *fcc*-structured binary alloys can be used as reliable models for modeling thermodynamic and structural properties of high-entropy alloys in a broad range of compositions. Phase instability upon heating and compression typical for high-entropy alloys with light metals such as Al, Co, Ni and Fe is not typical for refractory alloys based on platinum group metals. Platinum group metals are known as stable substances upon heating and compression. As soon as pure platinum metals and their binary alloys show extraordinary phase stability, their multicomponent alloys as well as high-entropy alloys did not show any phase transitions upon heating and compression, which makes them unique for high-temperature applications under extreme chemical impact and mechanical stress.

## 4. Conclusions

Single-source precursors strategy can be successfully applied for the preparation of high-entropy alloys of various compositions and structures. Double complex salts can be considered as effective single-source precursors for refractory multicomponent alloys. A systematic investigation of single-source precursors for binary alloys gives experimental evidence for synthetic possibilities to access multicomponent systems. Refractory alloys prepared using single-source precursors can be further applied as active elements of catalytic reactors, electrochemical and fuel cells [1,8,35,36]. Diverse single-source precursors of various chemical functionality, composition and stability should be designed to prepare useful functional alloys as nanostructured powders for catalytic applications. Based on the materials presented we could conclude the following:

(1) Using [Ir(NH_3_)_5_Cl][PtCl_6_], [Ir(NH_3_)_5_Cl][PtBr_6_], [Rh(NH_3_)_5_Cl]_2_[PtCl_6_]Cl_2_, [Rh(NH_3_)_5_Cl][PdCl_4_]·H_2_O and [Rh(NH_3_)_5_Cl][IrCl_6_] as single-source precursors, *fcc*–Ir_0.509(5)_Pt_0.491(5)_, *fcc*–Rh_0.657(4)_Pt_0.343(4)_, and *fcc*–Rh_0.497(5)_Pd_0.502(5)_ could be prepared by thermal decomposition in a hydrogen flow below 800 °C.

(2) Only *fcc*–Rh_0.66_Pt_0.33_ corresponded to the single-phase region on the phase diagram. *Fcc*–Ir_0.50_Pt_0.50_, *fcc*–Rh_0.50_Pd_0.50_ and *fcc*–Rh_0.50_Ir_0.50_ alloys corresponded to miscibility gaps on binary phase diagrams.

(3) Crystal structure and thermal decomposition in an inert atmosphere of bromide-containing salt [Ir(NH_3_)_5_Cl][PtBr_6_] were significantly different in comparison with isoformular chloride-based [Ir(NH_3_)_5_Cl][PtCl_6_]. Its thermal decomposition occurred at higher temperature with a formation of IrBr_3_ as a possible intermediate phase.

(4) Room temperature compression of *fcc*–Ir_0.50_Pt_0.50_, *fcc*–Rh_0.66_Pt_0.33_, *fcc*–Rh_0.50_Pd_0.50_ and *fcc*–Rh_0.50_Ir_0.50_ alloys up to 50 GPa did not reveal any phase transitions. Compressibility curves can be fitted using the third-order Birch-Murnaghan equation of state. For *fcc*–Ir_0.50_Pt_0.50_, *V*_0_/*Z* = 14.597(6) Å^3^·atom^−1^, *B*_0_ = 321(6) GPa and *B*_0_’ = 6(1); for *fcc*–Rh_0.66_Pt_0.33_, *V*_0_/*Z* = 14.211(3) Å^3^·atom^−1^, *B*_0_ =259(1) GPa and *B*_0_’ = 6.66(9) and for *fcc*–Rh_0.50_Pd_0.50_, *V*_0_/*Z* = 14.18(2) Å^3^·atom^−1^, *B*_0_ =223(4) GPa and *B*_0_’ = 5.0(3).

## Figures and Tables

**Table 1:** Preparatory conditions and crystallographic characteristics for *fcc*-structured Rh, Ir, Pt and Pd binary alloys.

Single-Source Precursor Ref.	Preparatory Conditions	Phase Composition	*a*, Å^*^	*V*/*Z*, Å^3^·atom^−1^	*Δa*, Å
[Ir(NH_3_)_5_Cl][PtCl_6_]	He flow, 400 °C	two *fcc* phases	*a_I_* = 3.919(6) *a_II_* = 3.847(6)	—	—
	H_2_ flow, 700 °C	*fcc*–Ir_0.50_Pt_0.50_ **sample A**	3.883(2)	14.537	−0.08
[Ir(NH_3_)_5_Cl][PtBr_6_]	He flow, 600 °C	two *fcc* phases	—	—	—
	H_2_ flow, 500 °C	*fcc*–Ir_0.50_Pt_0.50_	3.874(2)	14.535	−0.085
[Ir(NH_3_)_5_Cl]_2_[PtCl_6_]Cl_2_ [11] (only crystal structure was reported)	He flow, 460 °C	two *fcc* phases	*a_I_* = 3.855 *a_II_* = 3.917	—	—
	H_2_ flow, 500 °C	*fcc*–Ir_0.67_Pt_0.33_	3.870(1)	14.490	0.03
[Rh(NH_3_)_5_Cl][PtCl_6_]	He flow, 600 °C	*fcc*–Rh_0.72_Pt_0.28_ *fcc*–Rh_0.79_Pt_0.21_	*a_I_* = 3.837(4) *a_II_* = 3.828(4)	—	—
	H_2_ flow, 550 °C	*fcc*–Rh_0.5_Pt_0.5_	3.865(4)	14.434	0.002
[Rh(NH_3_)_5_Cl][PtBr_6_] [12]	He flow, 800 °C	*fcc*–Rh_0.38_Pt_0.62_ *fcc*–Rh_0.72_Pt_0.28_	*a_I_* = 3.878(3) *a_II_* = 3.836(3)	—	—
	H_2_ flow, 700 °C	*fcc*–Rh_0.50_Pt_0.50_	3.864(2)	14.423	0.001
[Rh(NH_3_)_5_Cl]_2_[PtCl_6_]Cl_2_ [13]	He flow, 460 °C	*fcc*–Rh_0.03_Pt_0.97_ *fcc*–Rh_0.93_Pt_0.07_	*a_I_* = 3.919(5) *a_II_* = 3.811(5)	—	—
	H_2_ flow, 500 °C	*fcc*–Rh_0.66_Pt_0.33_ **sample B**	3.845(5)	14.211	0.001
[Rh(NH_3_)_5_Cl][IrCl_6_] [5,14]	Ar flow, 550 °C	*fcc*–Rh_0.50_Ir_0.50_	3.817(2)	13.903	−0.004
	H_2_ flow 650 °C	*fcc*–Rh_0.50_Ir_0.50_	3.825(2)	13.991	0.004
[Rh(NH_3_)_5_Cl][IrBr_6_] [15]	He flow, 800 °C	*fcc*–Rh_0.50_Ir_0.50_	3.820(2)	13.936	0.001
	H_2_ flow, 600 °C	*fcc*–Rh_0.50_Ir_0.50_	3.824(2)	13.980	0.003
[Rh(NH_3_)_5_Cl]_2_[IrCl_6_]Cl_2_ [16]	He flow, 470 °C	*fcc*–Rh_0.66_Ir_0.33_	3.810(2)	13.827	−0.005
	H_2_ flow, 700 °C	*fcc*–Rh_0.66_Ir_0.33_	3.813(4)	13.859	−0.002
[Rh(NH_3_)_5_Cl][PdCl_4_]·H_2_O [9]	H_2_ flow, 400 °C	*fcc*–Rh_0.50_Pd_0.50_ **sample C**	3.845(4)	14.211	−0.002

* Cell parameters for pure metals: *a*(Pt) = 3.9231 Å (*V/Z* = 15.095 Å^3^); *a*(Ir) = 3.8394 Å (*V/Z* = 14.145 Å^3^); *a*(Rh) = 3.8031 Å (*V/Z* = 13.750 Å^3^) and *a*(Pd) = 3.8898 Å (*V/Z* = 13.819 Å^3^).

**Table 2:** Crystallographic characteristics for [*M*^I^(NH_3_)_5_Cl][*M*^II^*Г*_6_] *M*^I^ = Rh, Ir; *M*^II^ = Ir, Pt and *Г* = Cl and Br single-source precursors.

Composition	[Ir(NH_3_)_5_Cl][PtCl_6_] Powder, RT	[Ir(NH_3_)_5_Cl][PtBr_6_] Single Crystal, 150 K	[Rh(NH_3_)_5_Cl][IrCl_6_] Powder, RT	[Rh(NH_3_)_5_Cl][IrBr_6_] Single Crystal, RT	[Rh(NH_3_)_5_Cl][PtBr_6_] Single Crystal, RT
*a*, Å	11.568(3)	11.9099(13)	11.67(6)	12.030(6)	12.013(2)
*b*, Å	8.314(2)	8.3277(9)	8.348(7)	8.532(5)	8.401(2)
*c*, Å	16.104(3)	15.832(2)	15.65(3)	16.382(6)	15.999(3)
b, °	110.15(5)	90.000(4)	105.7(3)	106.23(1)	91.13(3)
*V*, Å^3^	1454.1	1570.3(3)	1468.0	1614.4	1614.4
Space group	*P*2_1_/*m*	*P*2_1_/*m*	*P*2_1_/*m*	*P*2_1_/*m*	*P*2_1_/*m*
*Z*	4	4	4	4	4
Molecular weight	720.62	987.32	628.45	895.15	898.01
*D,* g/cm^3^	3.287	4.176	2.844	3.683	3.70
PDF number	—	—	—	00-057-086501-080-8875	01-072-8177
ICSD number	—	1971298	—	421153	98115
Reference	present study	present study	[14]	[15]	[12]

**Table 3:** Parameters of the equations of state (EOS) for *fcc*-structured Ir, Rh, Pt and Pd binary alloys and their high-entropy alloys.

Composition (max. *P*)	*V*_0_/*Z*, Å^3^·atom^−1^ (*P* = 1 bar)^b^	*V*_0_/*Z*, Å^3^·atom^−1^ According to Zen’s Rule	*B*_0_, GPa *B*_0_’	*B*_0_, GPa According to Equation (2)	Ref.
*fcc*–Ir_0.42_Rh_0.58_ (up to 57 GPa)	13.90(8)	13.909	317(17) 6.0(5)	316.9	[5]
*fcc*–Ir_0.50_Pt_0.50_ (up to 15 GPa)	14.597(6)	14.625	321(6) 6(1)	304.7	**Sample A**
*fcc*–Pd_0.50_Rh_0.50_ (up to 45 GPa)	14.18(2)	14.224	223(4) 5.0(3)	225.7	**Sample C**
*fcc*–Pt_0.33_Rh_0.67_ (up to 47 GPa)	14.211(3)	14.180	259(1) 6.66(9)	292.1	**Sample B**
*fcc*–Ir (up to 67 GPa)	14.14(6)	―	341(10) 4.7(3)	―	[5]
*fcc*–Rh (up to 64 GPa)	13.73(7)	―	301(9) 3.1(2)	―	[5]
*fcc*–Pt *(up to 100 GPa)*	15.094(2)	―	277(2) 4.95(2)	―	[33]
*fcc*–Pd *(up to 100 GPa)*	14.718(2)	―	183 5.28	―	[34]
*fcc*–Ir_0.26_Os_0.05_Pt_0.31_Rh_0.23_Ru_0.15_ (up to 49 GPa)	14.16(9)	14.262	300(22) 6(1)	―	[5]
*hcp*–Ir_0.24_Os_0.21_Re_0.16_Rh_0.18_Ru_0.20_ (up to 45 GPa)	13.979(2)	13.882	317(2) 4.9(1)	―	[1]
